# Bone morphogenetic protein 2 promotes primordial follicle formation in the ovary

**DOI:** 10.1038/srep12664

**Published:** 2015-07-29

**Authors:** Prabuddha Chakraborty, Shyamal K. Roy

**Affiliations:** 1 Department of Cellular and Integrative Physiology, and Obstetrics and Gynecology; 2 University of Nebraska Medical Center, Omaha, NE.

## Abstract

Primordial follicles (PF) are formed when somatic cells differentiate into flattened pregranulosa cells, invaginate into the oocyte nests and encircle individual oocytes. We hypothesize that BMP2 regulates PF formation by promoting the transition of germ cells into oocytes and somatic cells into pregranulosa cells. E15 hamster ovaries were cultured for 8 days corresponding to postnatal day 8 (P8) *in vivo*, with or without BMP2, and the formation of PF was examined. BMP2 was expressed in the oocytes as well as ovarian somatic cells during development. BMP2 exposure for the first two days or the last two days or the entire 8 days of culture led to increase in PF formation suggesting that BMP2 affected both germ cell transition and somatic cell differentiation. Whereas an ALK2/3 inhibitor completely blocked BMP2-induced PF formation, an ALK2-specific inhibitor was partially effective, suggesting that BMP2 affected PF formation via both ALK2 and ALK3. BMP2 also reduced apoptosis *in vitro*. Further, more meiotic oocytes were present in BMP2 exposed ovaries. In summary, the results provide the first evidence that BMP2 regulates primordial follicle formation by promoting germ cell to oocyte transition and somatic cell to pre-granulosa cells formation and it acts via both ALK2 and ALK3.

Primordial follicle (PF) formation in the mammalian ovary occurs during fetal or postnatal period. This pool of resting follicles serves as the lifetime reserve for follicle recruitment during folliculogenesis in order to produce ovulatory follicles. Depletion of this population leads to menopause under normal condition and premature ovarian failure (POF) under pathological condition in women. POF can result in infertility and reduction in length and quality of normal reproductive life, along with several other devastating health conditions such as osteoporosis, cardiovascular diseases and cognitive disorders^1^. During fetal life, the process of follicle formation starts with the migration of primordial germ cells into the gonadal blastema, where they proliferate to form germ cell colonies known as egg-nests. Germ cells in the egg-nests enter meiosis and arrest at 1^st^ meiotic prophase to become the oocytes. Each colony is surrounded by undifferentiated somatic cells. Soon during ovary development, somatic cells differentiate into pregranulosa cells (preGCs), which invade the egg-nests to encapsulate individual oocytes to form PFs^2^. The communication between the oocytes and somatic cells plays an important role in oocyte survival and follicular development. Autocrine and paracrine interactions between follicular cells play major role in ovarian folliculogenesis^3^.

Bone morphogenetic proteins (BMPs) belong to the TGFβ superfamily of ligands and play important roles in cell proliferation and differentiation during development and in adult life^4^. TGFβ family members, such as growth and differentiation factor-9 (GDF9), BMP4, BMP7, BMP15, activin and inhibin have been shown to affect follicular development in mammalian ovaries^5^,^6^. BMP2, originally identified to be important for osteoblastic differentiation^7^,^8^, has been shown to play important role in neuronal^9^,^10^,^11^, and cardiomyocyte differentiation^12^. Recent evidence indicates BMP2 also induces cell motility and invasiveness in cancer cells, contractility in cardiomyocytes and self-expression in cardiomyocyte precursor cells. BMP2 has been reported to be expressed in the developing ovary and have role in primordial germ cell development^13^. BMP2 null mutation is embryonic lethal and fetuses contain low number of primordial germ cells^14^. BMPs utilize membrane bound heterodimeric complexes of type I and type II BMP receptors, among which either ALK2 or ALK3 or ALK6 acts as the type I receptor^15^. The canonical pathway for BMP2 signaling involves the heterodimerization of type I and type II BMP receptors for ligand binding followed by phosphorylation and activation of SMAD1/5/8. Either of the activated SMADs dimerizes with SMAD 4, translocates to the nucleus and modulates downstream gene expression^16^,^17^. ALK2 and ALK3 null mutation is embryo lethal^17^,^18^, while ALK6 null female mice show infertility due to a failure in ovarian cumulus cell expansion^14^. Because of these limitations, null mutations of BMP2-BMPR system cannot be used to reveal the role of BMP2 and its receptors in perinatal folliculogenesis. Consequently, the importance of BMP2 and the type of BMPRI utilized by BMP2 in the process of germ cell to oocyte transition and further primordial follicle formation remain unknown. We hypothesize that BMP2 regulates PF formation by promoting germ cell to oocyte transition and differentiation of ovarian somatic cells into preGCs leading to the formation of primordial follicles. The objective of the present study was to determine whether BMP2 stimulated primordial follicle formation in the developing hamster ovary by supporting the transition of germ cells to oocytes, and somatic cells to pregranulosa cells. The rationale for using golden hamsters in this study was that in contrast to mice or rats in which primordial follicles first appeared on postnatal day 1 and 4, respectively, morphologically distinct primordial follicles in developing hamster ovary were first visible on postnatal day 8. This long latency in PF formation in the hamster allows systematic and temporal intervention of PF formation without affecting maternal physiology. Further, in the hamster, the 8-day latency of PF formation *in vivo* is identical to that of *in vitro*^6^,^19^,^20^. Our preliminary data suggest that neither BMP4 nor BMP6 affect primordial follicle formation in the developing hamster ovary. Further, while BMP4 is expressed in neonatal hamster ovary, BMP6 is not. Based on this information, we selected to examine the effect of BMP2 on primordial follicle formation in the hamster.

## Results

### Expression of BMP2 mRNA and protein in the developing hamster ovary

**Figure 1:**
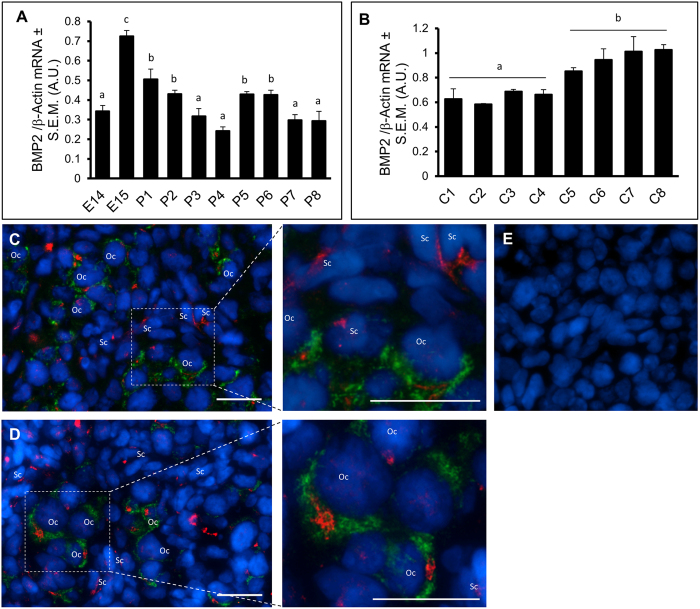
BMP2 mRNA and protein expression in hamster ovaries *in vivo* and *in vitro*. (**A**,**B**), quantitative RT-PCR analysis of BMP2 expression in perinatal hamster ovaries *in vivo* (**A**) and *in vitro* (**B**). Each bar represents the ratio of BMP2 and β-actin ± standard error of mean (s.e.m). P < 0.05, bars with different letter. *P* > 0.05, bars with same letter. (**C**,**D**), immunofluorescence localization of BMP2 protein in perinatal hamster ovary. Cytosolic expression of BMP2 (red) observed in the oocytes expressing GDF9 (green), and somatic cells during postnatal day 4 (**C**) and postnatal day 6 (**D**). Section incubated with the non-immune IgG and two second antibodies used in (**C**,**D**) as negative control. Nuclei were stained with DAPI (blue). Oc, Oocyte; Sc, Somatic cells. Scale Bar = 20 μM.

To determine whether BMP2 was expressed in the developing ovary and if the expression would correlate with the time of PF formation, total RNA from embryonic day 14 (E14) through postnatal day 8 (P8) ovaries were analyzed by qPCR for BMP2 and β-Actin mRNA. BMP2 transcript was expressed in the developing hamster ovary. The level of BMP2 mRNA increased significantly on E15, then declined before increasing again on P5 and P6. (Fig. 1A). The expression of BMP2 mRNA in ovaries *in vitro* remained mostly unchanged from C1 through C4 but started to increase from C5 through C8. Notably, BMP2 mRNA levels in cultured ovaries were higher than their *in vivo* counterparts (Fig. 1B). Immunofluorescence localization revealed cytosolic expression of BMP2 in both oocytes and somatic cells in the developing hamster ovary on P4 (Fig. 1C) and P6 (Fig. 1D). No immunosignal was present in sections incubated with non-immune IgG but with the same second antibody fluorophore (Fig. 1E).

### BMP2 stimulates primordial follicle formation *in vitro* during the early as well as late phases of perinatal ovary development

**Figure 2:**
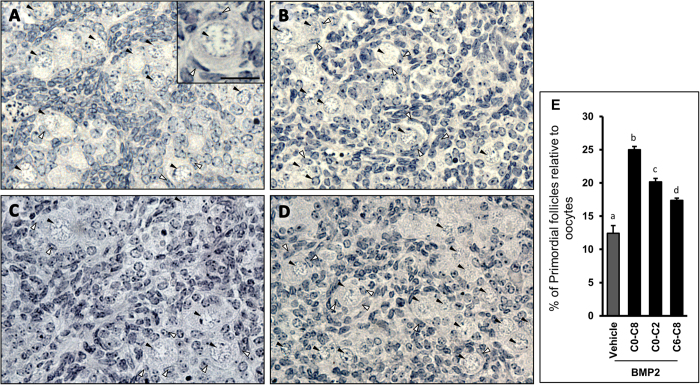
Primordial follicle formation in cultured hamster ovaries following BMP2 exposure. (**A**–**D**), representative sections of 8-day cultured ovaries (C8) exposed to vehicle (A) or BMP2 for 8 days (B) or to BMP2 for the first 48 h (**C**) or the last 48 h (**D**), showing the formation of primordial follicles (2A insert). Open arrowheads represent flattened granulosa cells, solid arrowheads represent oocytes. Scale Bar = 20 μM. (**E**), Index of primordial follicle formation upon either vehicle or BMP2 treatment during C0–C8 or C0–C2 or C6–C8. Each bar represents the PF index ± s.e.m. P < 0.05, bars with different letter.

To determine whether BMP2 affected the early or late phase of primordial follicle formation, E15 ovaries were exposed to BMP2 either for the first two days or the last two days or for 8 days in culture. After 8 days of culture, the first batch of primordial follicles appeared in the ovary (Fig. 2A,E). BMP2 exposure for the first two days led to a significant (*P* < 0.01) increase in PF index (Fig. 2C) compared with vehicle-treated group (Fig. 2A); however, it was significantly (P < 0.01) less than that was observed with 8 days of BMP2 exposure (Fig. 2B). The last two days of BMP2 exposure (Fig. 2D) also led to significant increase in PF formation compared to vehicle-treated control (*P* < 0.05), but the extent of increase was significantly less than those of either of the two BMP2 treated groups (Fig. 2E)

### BMP2 knockdown inhibits primordial follicle formation *in vitro*

**Figure 3:**
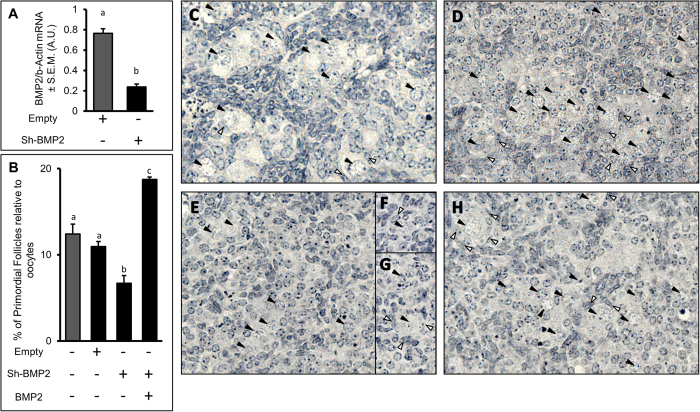
(**A**), BMP2 mRNA levels in C3 ovaries transduced with either pSiCoR-empty or pSiCoR-shBMP2 lentivirus on C0. Each bar represents the ratio of BMP2 and β-actin ± s.e.m in arbitrary units (A. U.). (**B**–**H**), PF index (**B**) and representative images (**C**–**H**) of ovaries following the exposure to vehicle (**C**), empty lentivirus (**D**), shBMP2 lentivirus (**E**–**G**) or shBMP2 lentivirus plus BMP2 (**H**). Scale Bar = 20 μM. Each bar represents the PF index ± s.e.m. For each graph, *P* < 0.05, bars with different letter. *P* > 0.05, bars with same letter.

To determine the role of endogenous BMP2 in PF formation, stable knockdown of BMP2 was achieved by transducing ovaries with lentivirus-shBMP2. To determine the specificity, three different shBMP2 targeting hamster BMP2 were used. All shBMP2 knocked down ovarian BMP2 >80%. However, none affected the expression levels of BMP4 and GDF9 transcripts, two closely related TGFβ family of proteins, in shBMP2 transduced ovarian RNA (data not shown), thus validating the on-target specificity of shBMP2. Ovarian BMP2 expression was knocked down in E15 ovaries with pSiCoR-shBMP2 lentivirus administered to the culture on day 0 of culture. Control groups received empty virus. BMP2 was administered after 48 hours. After additional 24 h culture, levels of BMP2 and β-actin mRNA levels were determined by RT-qPCR. A significant reduction in BMP2 mRNA level (p < 0.05) confirmed substantial decrease in BMP2 expression (Fig. 3A). Consequently PF index was reduced significantly (p < 0.05) after 8 days of exposure to shBMP2 (Fig. 3E–G) compared to either vehicle-treated (Fig. 3C) or empty lentivirus-treated (Fig. 3D) control groups. Notably, PF index was reduced by almost 50% compared to ovaries treated with empty virus (Fig. 3B). The decline in PF index was reversed when the decrease in endogenous BMP2 was overridden by exogenously added BMP2 (Fig. 3B,H).

### BMP2 acts via ALK2 and ALK3 to promote PF formation *in vitro*

**Figure 4:**
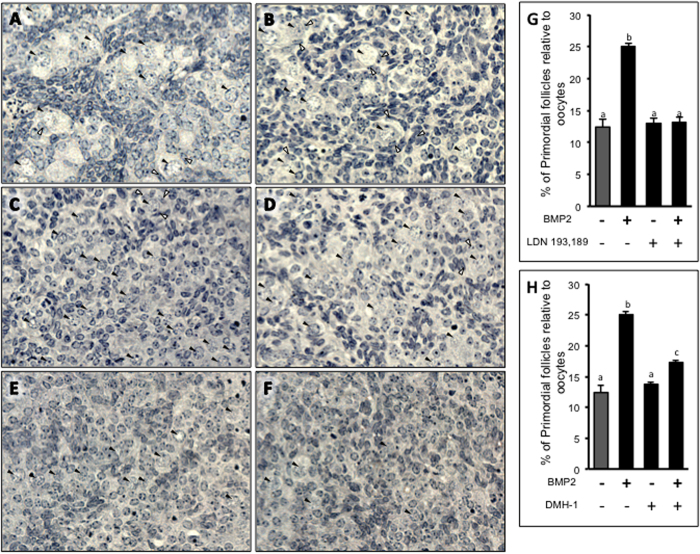
PF index following ALK2 and ALK3 inhibition in hamster cultured ovary. (**A**–**F)**, representative images of ovaries treated for 8 days with vehicle (**A**) or BMP2 (**B**) or LDN 193,189 (**C**) or LDN 193,189 plus BMP2 (**D**). Each bar represents the PF index ± s.e.m. B, ovaries were treated for 8 days with either vehicle or BMP2 or DMH1 or DMH1 plus BMP2. Each bar represents the PF index ± s.e.m. For each graph, P < 0.05, bars with different letter. *P* > 0.05, bars with same letter.

To determine which type I BMP receptor is involved in the BMP2-mediated PF formation process, either ALK2 was blocked by a specific inhibitor, DMH1 or both ALK2 and ALK3 were blocked by LDN193,189. BMP2 was added to the culture 1 h later, and the culture continued for 8 days. Neither LDN193,189 (Fig. 4C) nor DMH1 (Fig. 4E) alone affected PF index (Fig. 4G,H); however, while DMH-1 was partially effective in inhibiting BMP2-mediated increase in PF formation (p < 0.05) (Fig. 4F,H), ALK2/3 inhibitor LDN 193,189 completely blocked the BMP2 effect (Fig. 4D,G).

### BMP2 inhibits apoptosis in the developing ovary

**Figure 5:**
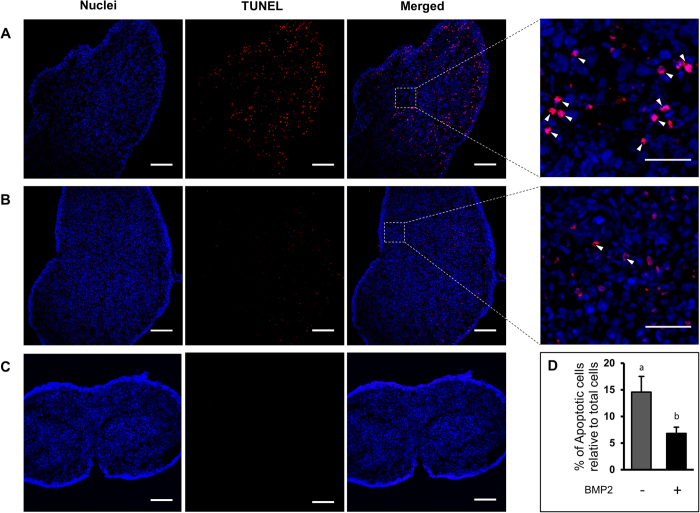
Apoptotic cell death in C3 ovaries cultured with either vehicle or BMP2. (**A**,**B**), TUNEL labeling (red) in C3 ovaries either treated with vehicle (**A**) or BMP2 (**B**). C, negative control. Nuclei are DAPI positive (blue). Bar = 100 μM. Inset, magnified view of TUNEL positive germ cells (open arrowheads). Bar = 25 μM. D, ratio of apoptotic cells to total cells. Each bar represents the percentage of apoptotic cells relative to total cells ± s.e.m. P < 0.05, bars with different letter.

**Figure 6:**
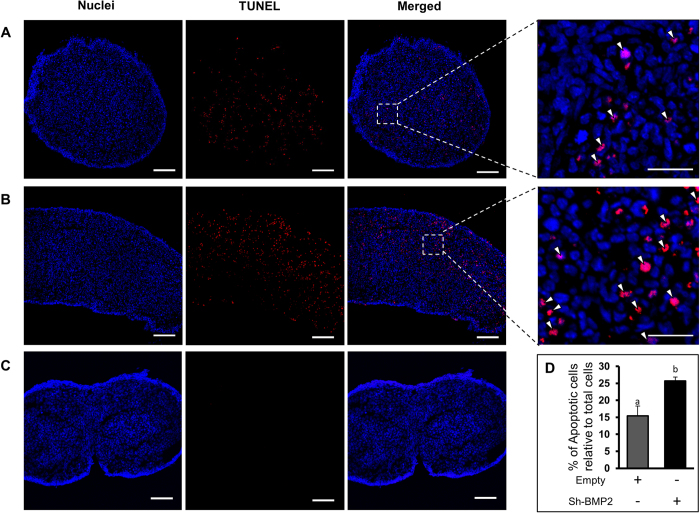
(**A**,**B**), TUNEL labeling (red) in C4 ovaries either treated with empty (**A**) or shBMP2 (**B**). (**C**), negative control. Nuclei are DAPI positive (blue). Bar = 100 μM. Inset, magnified view of TUNEL positive germ cells (open arrowheads). Bar = 25 μM. D, ratio of apoptotic cells to total cells. Each bar represents the percentage of apoptotic cells relative to total cells ± s.e.m. P < 0.05, bars with different letter.

To determine if BMP2 prevented apoptotic cell death in the developing ovary, DNA fragmentation in vehicle or BMP-2 treated ovaries was determined by TUNEL staining. Significant decrease in TUNEL staining was visible within 3 days of culture with BMP2 compared to vehicle-treated control (Fig. 5A,B,D), and majority of the TUNEL positive cells were germ cells. No immunosignal was present in negative control sections (Fig. 5C). Upon BMP2 knockdown, a significant increase in the TUNEL staining was found after 4 days of culture (Fig. 6A,B,D). No immunosignal was present in negative control sections (Fig. 6C).

### BMP2 treatment increases the meiotic entry of the oocytes

**Figure 7:**
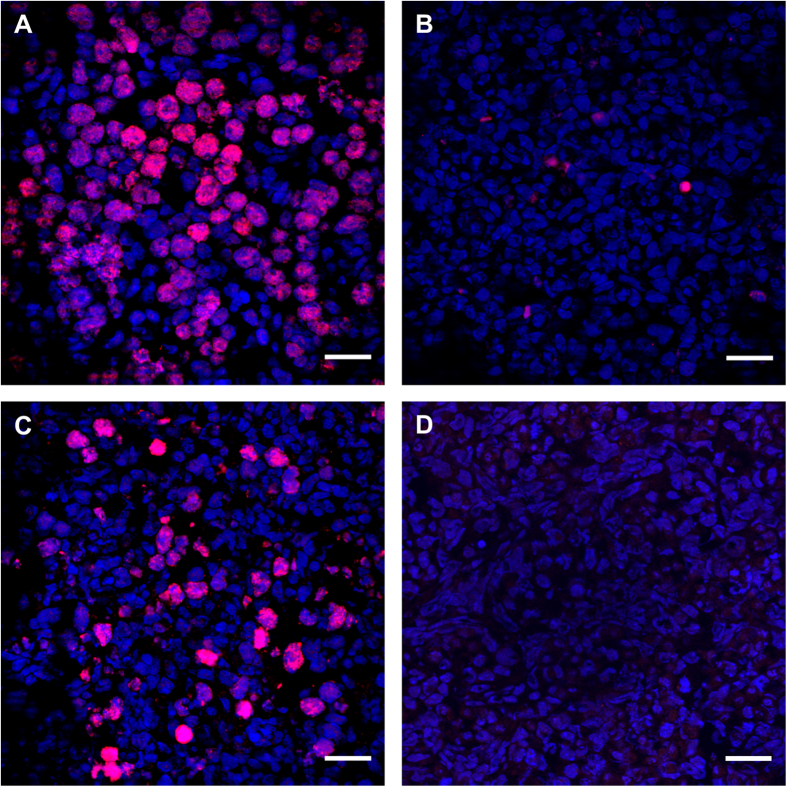
Immunolocalization of g (gamma)His2A.X (red) in A, P1 ovary; B, E15 ovaries cultured for 24h (C1) with vehicle or C, with BMP2. D, antibody control. A P1 ovary section incubated without the antibody. Nuclei are DAPI positive. Bar=20u (micro)M.

**Figure 8:**
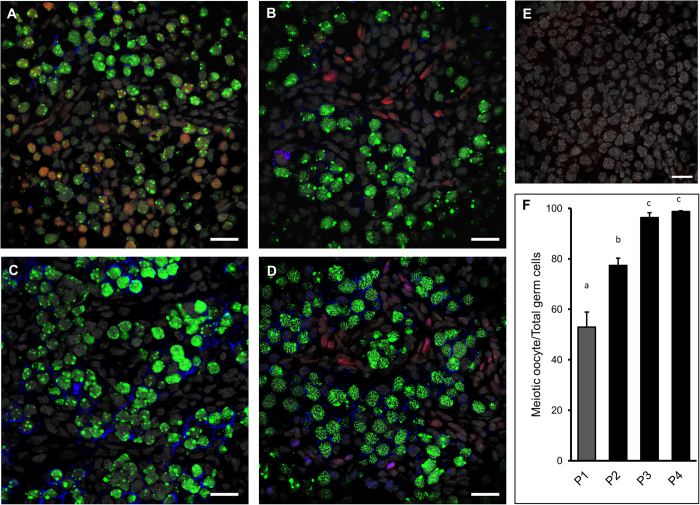
Meiotic entry of germ cells in developing hamster ovaries. Immunolocalization of SYCP3 (green), MCM2 (red) and MVH (blue) positive germ cells to determine the abundance of proliferating or meiotic germ cells in ovaries from P1 (**A**); P2 (**B**); P3 (**C**) and P4 (**D**) ovaries. Ovary sections incubated with non-immune IgG along with three second antibodies used in (**A**–**C**) and DAPI as negative control (**E**). Nuclear DAPI signal was kept as gray to avoid confusion with MVH pseudocolor. Bar = 20 μM. (**F**), quantitation of the meiotic oocyte index in P1–P4 ovaries. Each bar represents the percentage of meiotic oocytes relative to total germ cells ± s.e.m. *P* < 0.05, bars with different letter. *P* > 0.05, bars with same letter.

**Figure 9:**
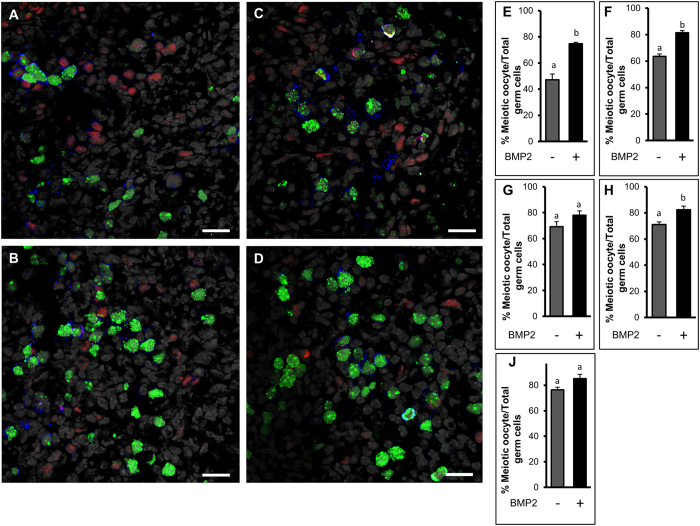
Meiotic entry of germ cells in cultured hamster ovaries. Immunofluorescence analysis of cultured ovaries to determine meiotic entry of oocytes upon BMP2 exposure on P3 (**A**,**B**) and P4 (**C**,**D**) treated with either vehicle (**A**,**C**) or BMP2 (**B**,**D**) and probed for SYCP3 (green), MCM2 (red) and MVH (blue). Nuclei are gray. Negative controls were exactly the same as Fig. 8E (data not shown). (**E**–**J**), Quantitation of the meiotic oocyte index on C3 through C7, respectively. Each bar represents the percentage of meiotic oocytes relative to total germ cells ± s.e.m. *P* < 0.05, bars with different letter. *P* > 0.05, bars with same letter. Bar = 20 μM.

**Figure 10:**
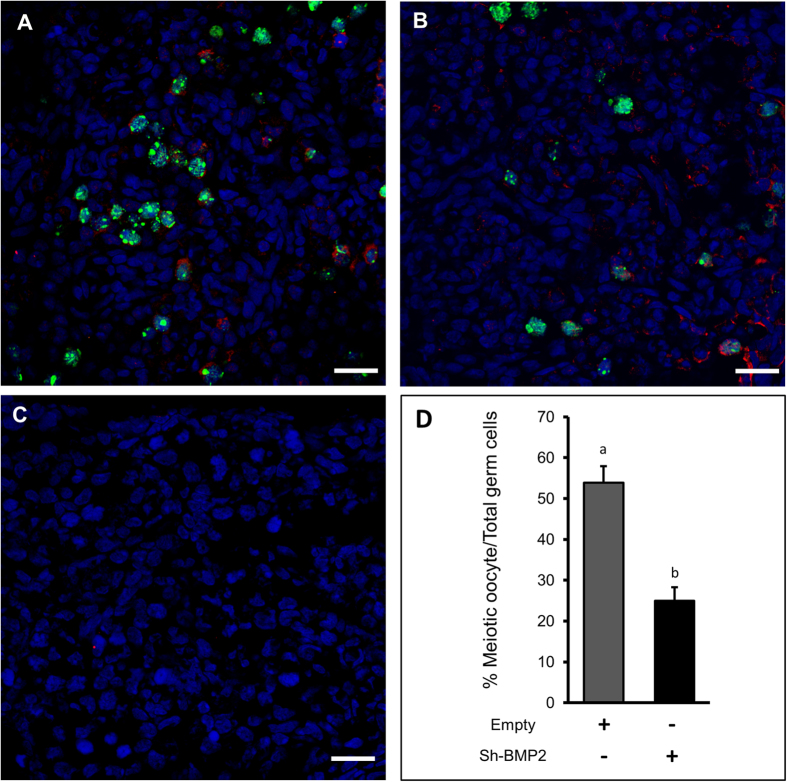
Meiotic entry of germ cells in cultured hamster ovaries following BMP2 knockdown. Immunofluorescence analysis of cultured ovaries on C4 treated with empty (**A**) or shBMP2 (**B**), and probed for SYCP3 (green) and MVH (blue). Antibody negative control for SYCP3 immunostaining (**C**). Quantitation of the meiotic oocyte index (**D**). Each bar represents the percentage of meiotic oocytes relative to total germ cells ± s.e.m. P < 0.05, bars with different letter. Bar = 20 μM.

Immunostaining for γHis2A.X, which was used to detect double-strand break during the initiation of meiosis, revealed an increased presence of γHis2A.X–positive germ cells in BMP2-treated cultured ovaries on C1 (Fig. 7A) compared with vehicle-treated control (Fig. 7B). Similar increase in γHis2A.X positive germ cells was noted *in vivo* in P1 ovary (Fig. 7C); however, no immunosignal was present in the negative control (Fig. 7D). To examine germ cell to oocyte transition *in vivo* and *in vitro* the expression of SYCP3 (a marker for meiosis), MCM2 (a proliferation marker) and MVH (an oocyte marker) were examined either in *in vivo* developing ovaries from P1 through P4 (Fig. 8A–D) or in ovaries cultured with or without BMP2 on C3 and C4 (Fig. 9A–D). The number of oocytes showing characteristic SYCP3 staining of meiotic prophase were calculated and normalized to total number of germ cells. The results were defined as the meiotic entry index. The meiotic entry index in ovaries increased from P1 through P3 and reached near 100% on P4 *in vivo* (Fig. 8F). Negative control did not show any immuostaining (Fig. 8E) indicating the specificity of the antibodies. In cultured ovaries, the meiotic entry index for BMP2-treated ovaries were significantly higher (p < 0.05) compared to that of vehicle-treated control group on culture days 3 (Fig. 9E) and 4 (Fig. 9F). Over the span of culture, the difference in meiotic entry index between BMP2 treated versus untreated declined (Fig. 9G–J), which was most likely due to the effect of endogenous BMP2. Cell proliferation index remained unchanged (data not shown). SYCP3 and MVH were also localized in cultured ovaries in which BMP2 expression was knocked down with shRNA (Fig. 10A,B). The meiotic entry index in BMP2 knockdown ovaries was significantly less (p < 0.05) than ovaries treated with the empty virus (Fig. 10D). Further, negative control was devoid of immunosignal (Fig. 10C).

## Discussion

The present study indicates that developmentally regulated BMP2 mRNA is spatiotemporally expressed in the developing hamster ovary both *in vivo* and *in vitro*. Bone morphogenetic proteins are members of TGFβ superfamily of proteins and play important role in ovarian folliculogenesis. BMP2 is a secreted protein, which appears as early as E7.5 in mouse embryos^19^ and expressed in many tissues during embryonic development^20^,^21^. BMP2 mRNA expression in the mouse embryonic ovary has been detected from E 11.5 onwards and the expression peaks on E13.5^22^. BMP2 mRNA expression in human developing ovaries occurs during 8–9 weeks, and increases rapidly through 14–16 weeks^23^. The increase in BMP2 mRNA in the hamster ovary around the time of germ cell to oocyte transition and before the formation of PFs suggests that BMP2 may affect early oocyte development. Further, the rise in BMP2 expression during P5 and P6 indicates that BMP2 may also affect somatic cell differentiation and PF formation *in vivo*. The exact reason for the gradual increase in BMP2 mRNA expression in cultured ovaries from C4 is not known, but it may be due to the lack of physiological regulation in organ cultures. However, gremlin mRNA expression corresponds to BMP2 mRNA expression (data now shown). Therefore, it is logical to speculate that intraovarian mechanism maintains functional BMP2 levels. BMP2 is primarily expressed by the somatic cells in the developing ovary^24^. However, the expression of BMP2 protein in both somatic cells and oocytes in the egg nests in the present study suggests that BMP2 may differentially control the functions of germ cells and somatic cells.

The results indicate that BMP2 promotes PF formation by acting through both ALK2 and ALK3. Among the three type I receptors for BMPs, ALK2 mRNA is expressed in the mouse ovary as early as E13 and the expression continues until birth^25^. ALK3 mRNA expression is detected in the mouse ovary from E11.5^22^ and in the hamster ovary from E13^26^. ALK3 expression is upregulated in the early and in the late phases of postnatal hamster ovary development^26^. ALK6 mRNA is also expressed in the hamster ovary from E13; however, the expression level is lower than that of ALK3 until P6^26^. BMPs are known to bind different type I receptors with varied affinity in different cell types. BMP2 generally binds to ALK3^27^, whereas BMP4 has been shown to act via both ALK2 and ALK6^27^,^28^. However, these BMPs generally use BMPRII as the type II receptor that separates them from other TGFβ family members such as activins^27^. The action of BMP2 on PF formation seems to be mediated by both ALK2 and ALK3. Because concurrent blockade of ALK2 and ALK3 completely prevents BMP2-mediated increase in PF formation, it is unlikely that ALK6 plays a major role in BMP2 signaling in this phase of follicle development.

The increased PF index due to BMP2 exposure during the first 48 h of culture seems primarily due to increased oocyte development. Conversely, increased PF index after majority of germ cells have differentiated into oocytes suggests strongly that BMP2 facilitates germ cells to oocytes transition at the early phase and induces somatic cell differentiation at the later phase of ovary development in order to synchronize oocyte and preGC communication. In addition, an increase in the number or quality of viable oocytes may also contribute to the process. However, increased number of oocytes alone is inadequate to increase PF index; therefore, it stands to reason that BMP2 also induces the differentiation of somatic cells to preGCs. The specificity of BMP2 effect on PF index is apparent from the knockdown of BMP2 expression and its reversal by BMP2.

Germ cells undergo proliferation in the hamster developing ovary mostly from E14 through P1. Following proliferation, germ cells enter meiotic prophase I and remain arrested at the deplotene stage in the mouse ovary^29^. During meiotic transition, the lateral-element proteins SYCP2 and SYCP3 are recruited to help forming the synapsis between homologous chromosomes^30^,^35^. Therefore, the presence of SYCP3 in condensed chromosomal DNA marks the entry of germ cells to meiotic prophase I^36^. Another marker of meiotic entry, γH2A.X is associated with the double-strand break during the entry into meiosis^37^. Both of these markers are upregulated by BMP2 treatment in cultured hamster ovaries indicating that BMP2 promotes the entry of germ cells into meiotic prophase. The gradual increase in meiotic entry index in the control group with the extension of culture days suggests that endogenously produced BMP2 slowly but steadily upregulates this vital process. This conjecture is supported by a drastic decline in the meiotic oocyte index following BMP2 knockdown. The dramatic effect of BMP2 during the first 3 days of culture and a marked reduction in the meiotic oocyte index after BMP2 knockdown highlight the importance of BMP2 in regulating the meiotic entry. STRA8, which is an upstream factor, regulates the initiation of the meiotic prophase by controlling the pre-meiotic DNA replication^34^,^38^. BMP2 has been reported to stimulate the expression of MSX1, which controls STRA8 expression in the developing mouse ovary^39^. These lines of evidence suggest that BMP2 promotes meiotic oocyte formation by regulating the molecular process of meiotic initiation in the hamster ovary.

During normal development, the majority of germ cells die by apoptosis^40^ due to various reasons^41^,^42^. Therefore, it seems logical that there will be less apoptotic oocytes when more germ cells undergo uninterrupted meiotic entry. BMP2 has been shown to stimulate follistatin expression in the developing mouse ovary^22^. Follistatin supports germ cell survival^43^. Therefore, the maintenance of germ cell viability by BMP2 in hamster ovaries as evident in the present study will result in more healthy oocytes; consequently, more primordial follicles.

In summary, BMP2 promotes the development of primordial follicles by acting through either ALK 2 or ALK3 in hamster fetal ovaries *in vitro*. It is evident that BMP2 promotes the process of primordial follicle formation by acting on both oocyte development and somatic cell differentiation. Further, BMP2 promotes meiotic entry of oocytes *in vitro* and prevents germ cell apoptosis resulting in the availability of healthier pool of oocytes for primordial follicle formation.

## Materials and Methods

Antibodies to BMP2, SYCP3, MVH, γHis2A.X and MCM2 were obtained from Abcam (Cambridge, MA). Alexa-conjugated secondary antibodies were obtained from Invitrogen (Carlsbad, CA). Plastic embedding medium was obtained from Electron Microscopy Sciences (Hatfield, Pa). Tissue-Tek Optimal Cutting Temperature (OCT) compound was obtained from Sacura Finetek USA Inc. (Torrance, CA). Phenol red-free DMEM, linolenic acid, BSA, and fine chemicals were purchased from Sigma Chemical Company (St. Louis, MO). LDN 193,189 was purchased from Sigma Chemical Co. (St. Louis, MO). DMH1 was purchased from Tocris Bioscience (Bristol, UK). Human holo-transferrin was obtained from BD pharmaceuticals (San Jose, CA). Quantitative RT-PCR primers and probes were synthesized by Sigma Chemical Company. RNeasy mini kit and Taq DNA polymerase were from Qiagen, Inc. (Valencia, CA). The terminal deoxynucleotidyl transferase-mediated deoxyuridine triphosphate nick-end labeling (TUNEL) kit was from Roche pharmaceuticals (Mannheim, Germany). All other molecular biology grade chemicals were obtained from Sigma Chemical Company or Fisher Scientific (Pittsburgh, PA).

### Animals and treatments

Female golden hamsters (*Mesocricetus auratus*) (90–100 g) were obtained from Harlan Sprague Dawley Laboratories and housed in a climate-controlled environment with 14 h light and 10 h dark cycle, and fed ad libitum according to the UNMC Institutional Animal Care and Use Committee and United States Department of Agriculture guidelines. The use of hamsters in the research was approved by UNMC IACUC. Females with at least three consecutive estrous cycles were mated with males in the evening of proestrus, and the presence of sperm in the vagina the next morning was considered day 1 of pregnancy. Hamster gestation lasts for 16 days, and pups are born on 16th day of gestation, which we considered the first day of postnatal life. Ovaries from embryos and postnatal pups were collected and embedded in OCT compound for frozen sections or flash-frozen in liquid N_2_ for total RNA extraction.

### Assessment of BMP2 effect on primordial follicle formation *in vitro*

Ovaries were collected between 0800 and 1000 hours from embryonic day 15 (E15) hamsters in phenol red-free DMEM containing antibiotics at room temperature, cleaned of adherent tissues, and cultured in the presence of 0.1 μg/ml insulin, and 1.25 μg/ml transferrin, 1.25 μg/ml selenium, and 10.7 μg/ml linoleic acid (ITS; as described previously)^44^,^45^ with or without either 50 ng/ml of BMP2 (R & D Systems, Minneapolis, MN) or 500 nM LDN 193,189 (ALK2/3 inhibitor) or 200 nM DMH1 (ALK2 inhibitor). All ovaries were placed in culture by 1300 hours of the day of collection. Ovaries in culture were pretreated with inhibitors for 1 hour before addition of ligands and medium was changed every 48 hours. For each group, there were at least three E15 ovaries from three pregnant hamsters. Ovaries were retrieved at 1200 hours after 8 days of culture and processed for morphometric evaluation of the formation and development of primordial follicles. For morphometric evaluation of primordial follicle formation, total number of primordial follicles and oocytes regardless of their association with somatic cells in each section were counted. Then the percentage of primordial follicles with respect to the oocytes was determined. The percentage was considered as the primordial follicle (PF) index. Values from at least three ovaries were used to calculate the mean + standard error of mean (s.e.m). This was necessary because primordial follicles were the only follicles in the ovary at the end of culture.

### Immunofluorescence Localization of BMP2, γHis2A.X, SYCP3, MVH, GDF9 and MCM2

Frozen ovary sections (6 μm thick) were fixed in freshly prepared 4% paraformaldehyde in PBS (pH-7.4) at 4 °C and washed 3 times 5 minutes each with PBS. The sections were treated with Image-iT FX signal enhancer for 20 minutes and followed by 1 hour of blocking with 10% normal donkey serum at room temperature. Next, the sections were incubated with either SYCP3 or MCM2 or MVH or GDF9 or γHis2A.X or BMP2-specific antibody overnight at 4 °C. The signal was developed using donkey second antibodies conjugated with either alexa-fluor 488 or alexa-fluor 594 or alexa-fluor 647. Nuclei were stained with 4′,6-diamino-2-phenylindole (DAPI). The images were captured by either a Leica Confocal microscope (North Central Instruments, Plymouth, MN) and Leica LAS image analysis software or Leica DM research microscope (North-Central Instruments, Inc.; Plymouth, MN) equipped with Qimaging Retiga digital camera (Surray, Canada) using Openlab software (Improvision, Lexinton, MA).

### Measurement of BMP2 and β-Actin mRNA levels

Ovarian total RNA was used for qPCR determination of the levels of respective mRNAs using real-time PCR primer pairs and fluorescence-labeled probes (Table 1) in a Chromo4 real-time thermocycler (Bio-Rad). cDNA standards were generated from *in vitro*-transcribed pure mRNA for each gene and were amplified along with the sample and the levels of mRNA were determined from the standard curve.

### Lentiviral delivery of shRNA construct in ovaries *in vitro*

BMP2 shRNA duplex was cloned into pSiCoR vector (Addgene, Cambridge, MA) and lentivirus particles were prepared. E15 ovaries were exposed to lentivirus containing medium with polybrene for 6 hours and then cultured with either vehicle or with BMP2 for 8 days.

### Statistical analysis

Ovaries were collected from at least three animals for each group for all experiments. All quantitative data were analyzed by either unpaired Student’s t-test or one-way ANOVA with Tukey’s *post hoc* test for multiple comparisons using GraphPad Prism 5 software (Graph Pad software Inc., La Jolla, CA). The level of significance was *P* < 0.05.

## Additional Information

**How to cite this article**: Chakraborty, P. and Roy, S. K. Bone morphogenetic protein 2 promotes primordial follicle formation in the ovary. *Sci. Rep*. **5**, 12664; doi: 10.1038/srep12664 (2015).

## Figures and Tables

**Table 1:** Sequences of BMP2 shRNA and β-Actin primers and probes.

Gene	Sequence
BMP2	F: 5′-GACACCAGGTTAGTGAATCAGAACA-3′
	R: 5′-TCTTGGAGACACCTGGCTTCTC-3′
	Probe: 6-FAM-5′-TGCTGTGATGCGGTGGACTGCA-3′-BLACKHOLE
β-Actin	F: 5′-TGACCGAGCGTGGCTACAG-3′
	R: 5′-CTTCTCTTTGATGTCACGCACAAT-3′
	Probe: 6-FAM-5′-TCACCACCACAGCCGAGAGGGA-3′-BLACKHOLE
Hamster shBmp2 sense	[phos]TGCAGGTCTTTGCACCAAGATTCAAGAGATCTTGGTGCAAAGACCTGCTTTTTTC
Hamster shBmp2 antisense	[phos]TCGAGAAAAAAGCAGGTCTTTGCACCAAGATCTCTTGAATCTTGGTGCAAAGACCTGCA

(Chakraborty and Roy)
